# Strengthening health financing at sub-national level in Kenya: a stakeholder and needs mapping through a mixed methods approach

**DOI:** 10.11604/pamj.2024.48.186.44484

**Published:** 2024-08-27

**Authors:** Alex Olateju Adjagba, James Odhiambo Oguta, Elvis Omondi Wambiya, Catherine Akoth

**Affiliations:** 1 University of Western Cape, Cape Town, South Africa,; 2 UNICEF Zimbabwe Country Office, Harare, Zimbabwe,; 3 UNICEF Eastern and Southern Africa Regional Office (ESARO), Nairobi, Kenya,; 4 Sheffield Centre for Health and Related Research, School of Medicine and Population Health, University of Sheffield, Sheffield, South Yorkshire, England

**Keywords:** Public financial management, health financing, technical assistance, technical support, stakeholder analysis, partner mapping, county, Kenya

## Abstract

**Introduction:**

health financing aims to ensure that the overall goal of the health system is attained. Countries with decentralised healthcare systems such as Kenya, face further challenges due to limited public financial management capacity within sub-national governments. While partner support has proved impactful in addressing these challenges, there is a paucity of evidence on the nature and distribution of the support in Kenya. This study sought to examine the current technical support and health financing support offered by partners across the 47 counties in Kenya.

**Methods:**

the study used a descriptive cross-sectional design with a mixed methods approach. Quantitative data were collected from organisation representatives using semi-structured questionnaires and analysed using Microsoft Excel. Qualitative data were collected through key informants and in-depth interviews involving county Department of Health officials in 15 counties in Kenya. Interview recordings were transcribed and thematically analysed using NVIVO version 14.

**Results:**

twenty (20) organisations reportedly provided health financing support to counties with planning, budgeting and health financing advocacy being the most supported work streams by partners. While each county had more than one partner supporting health financing activities, the western counties had more partners compared to other regions of Kenya. Whereas partner support was well acknowledged at the county level, there was a lack of coordination and alignment of partner activities with county priorities.

**Conclusion:**

these findings highlight the essential need for national governments to ensure effective coordination of the technical assistance provided by partners to subnational levels and to ensure equitable distribution of support and alignment with county health priorities and needs.

## Introduction

Health financing is the mobilisation and allocation of funds to cover the health needs of individuals, countries, and health systems [1]. All aspects of healthcare, from infrastructure, personnel and quality of care are influenced by the way the health system is financed [1]. Africa has the lowest health spending per capita globally [2]. The inadequate financing for health is characterised by relatively high out-of-pocket expenditure and over-reliance on unpredictable donor funding [2]. Out-of-pocket spending as a percentage of total health expenditure exceeds 20% in more than 80% of African countries [2]. The Abuja declaration on allocation of at least 15% of the national budget to health remains a mirage for most countries [3]. Several factors have contributed to this situation, including poor planning, lack of accountability and transparency, and poor partner support and coordination [4].

Decentralisation has increasingly been adopted as a governance reform by different countries to increase the reach of government services to local communities [5,6]. Decentralisation takes different forms, ranging from deconcentration, where national government departments provide lower-level functions, to devolution, involving both political and administrative transfer of functions to autonomous local governments [7]. Kenya adopted a devolved system of government in 2013, which resulted in the decentralisation of other services including health to the 47 semi-autonomous county governments [8]. The national government is responsible for formulating health policies and provides oversight over national referral health facilities, while county governments provide primary healthcare services from the community level to county referral health facilities [9,10]. The devolved health system faced a myriad of challenges relating to staffing, inadequate equipment and medical supplies which affected service delivery [11]. Additionally, public players in the county governments were faced with inadequate capacity to manage the health system in counties [8,12].

In Kenya, the health sector is financed through different sources including government budget allocation, private institutions, health insurance, donor funding [13] and households [14]. The county health system is financed through allocations from the national government budget, own source revenues from facilities and funding from donors [15]. Health financing in Kenya is facing challenges such as low awareness and inadequate capacity in health financing and public financial management [8]. Though inadequately funded, budget allocation to health is often underspent due to disbursement delays occasioned by poor capacity and understanding of public financial management procedures [16]. These challenges necessitate technical support from a wide range of stakeholders.

Different partners support the Kenyan health sector at different levels and capacities. The Kenya Health Sector Partnership and Coordination Framework set out mechanisms for partnerships between all stakeholders to align their work towards the same goals [17]. The participation of different stakeholders in health financing in Kenya has been established [18], with the private sector being the greatest health financing agent for county governments [9]. However, there is a need for more evidence on the nature and distribution of that support. This study sought to map health financing partners at the county level and assessed experiences and perceptions of health financing support at the county level. Understanding the role of each stakeholder in financing is critical to improving efficiency in the health system [19].

## Methods

**Study design:** this study adopted a mixed methods design involving the quantitative characterization of stakeholder organisations and qualitative interviews with county health officials.

**Study setting:** Kenya is a lower middle-income country located in East Africa, bordering Uganda to the west, Somalia to the East, Ethiopia to the north and Tanzania to the south. Kenya moved from eight regions/provinces to a devolved system of government composed of one central government and 47 semi-autonomous county governments. The county governments are responsible for overseeing health service delivery at the county with each county having a department of health that conducts planning and implementation of health service delivery within the county level.

**Sampling:** we conducted a desk review to identify the list of potential stakeholders for inclusion in the mapping. Organisations targeted were those that work in health financing-related areas in Kenya. The organisations were identified through an internet search, a review of Ministry of Health documents, and chain referrals. Twenty-eight (28) organisations were identified through this process and contacted. Additionally, fifteen counties were purposively sampled to obtain perspectives of county health officials on support received by counties from different stakeholders. The 15 counties selected for the qualitative study were Bomet, Bungoma, Kakamega, Kiambu, Kisii, Kisumu, Makueni, Mombasa, Muranga, Nairobi, Nakuru, Taita Taveta, Tharaka Nithi, Uasin Gishu and West Pokot. Participants at the county level were also purposively selected based on their roles within the Department of Health with a snowballing approach used to further identify relevant participants.

**Participants:** a total of 77 senior county officials were selected and interviewed.

**Data collection:** quantitative data were collected with the aid of a semi-structured questionnaire seeking to understand the role of organisations in health financing and their scope of work at the counties. The questionnaires were either self-administered or interviewer-administered, depending on the preference of the respondents. Subsequently, qualitative data were collected through key informant interviews and in-depth interviews conducted with 77 senior county health officials in the 15 counties, using semi-structured interview guides.

**Data analysis:** quantitative data from desk reviews, interviews and questionnaires were summarised and analysed using Microsoft Excel (Ms Excel 2016) and presented in the form of tables, graphs and maps. The audio recordings of the interviews were transcribed with the support of two research assistants. To ensure trustworthiness, the four researchers read the transcripts to understand and familiarise with the depth of the data and emerging themes. Two members of the research team then independently coded ten initial transcripts (five each), on NVIVO version 14. The team met to compare the emerging codes and prepared a final codebook that was applied to the remaining transcripts. Additional codes were iteratively added as nuanced during the coding process. All related codes were grouped into sub-themes, leading to interpretable themes.

**Ethics statement:** ethical review and approval for this study was granted by Moi Teaching and Referral Hospital- Moi University Institutional Research and Ethics Committee (approval number 003605). Participants from the organisations provided either written or oral informed consent depending on whether the interview was on the phone or face-to-face. Participants from the counties provided written informed consent before the interviews. The team anonymised all transcripts before analysis and reporting.

## Results

### Quantitative findings

**Figure 1:**
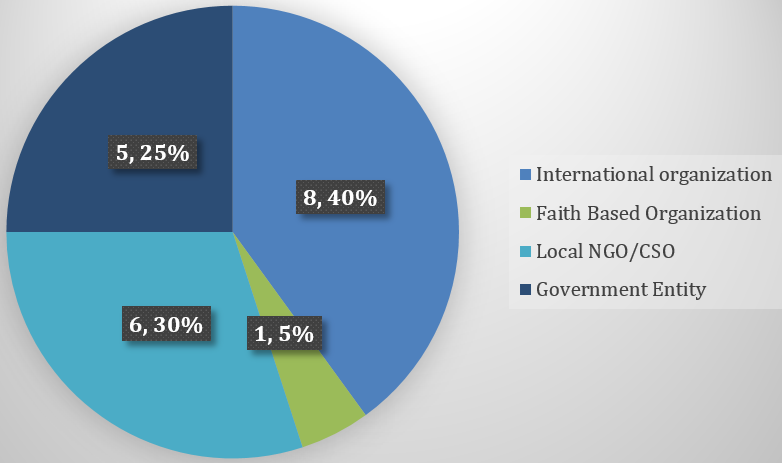
type of organization providing health financing support

**Organisations providing health financing support in Kenya:** out of the initial twenty-eight (28) organisations identified and contacted, we received responses from twenty (20). Telephone and face-to-face interviews were conducted for eleven (11) organisations, while nine (9) organisations responded to the online questionnaire. Most Organisations (15) were non-governmental, with nine being international and six local ones. Five government entities, including two research institutions, participated in the study (Figure 1).

**Figure 2:**
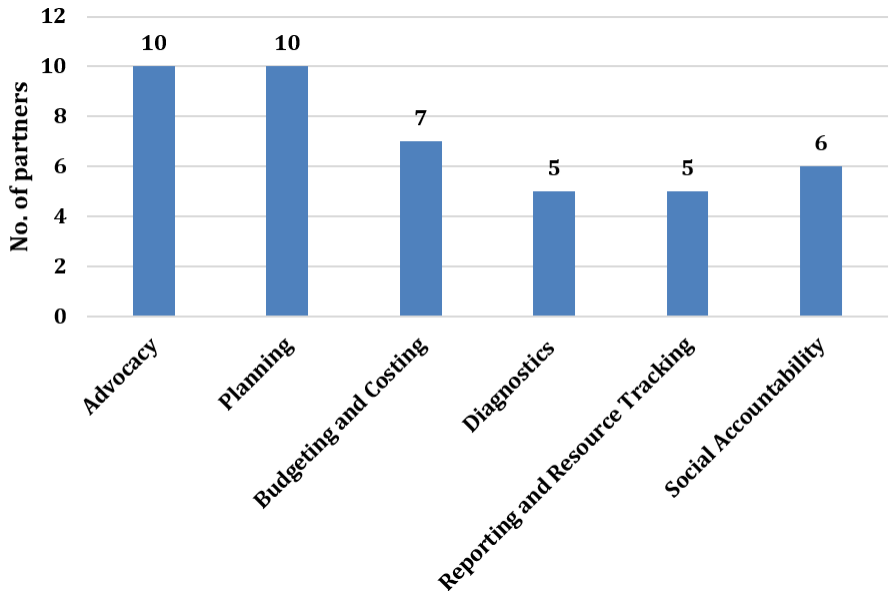
health financing work streams supported by partners

**Health financing work-streams supported by partners:** ten (10) organisations supported health financing advocacy and health planning, and seven supported county-level budgeting and costing. Health financing diagnostics and reporting, and resource tracking had the least number of partners involved (Figure 2).

**Figure 3:**
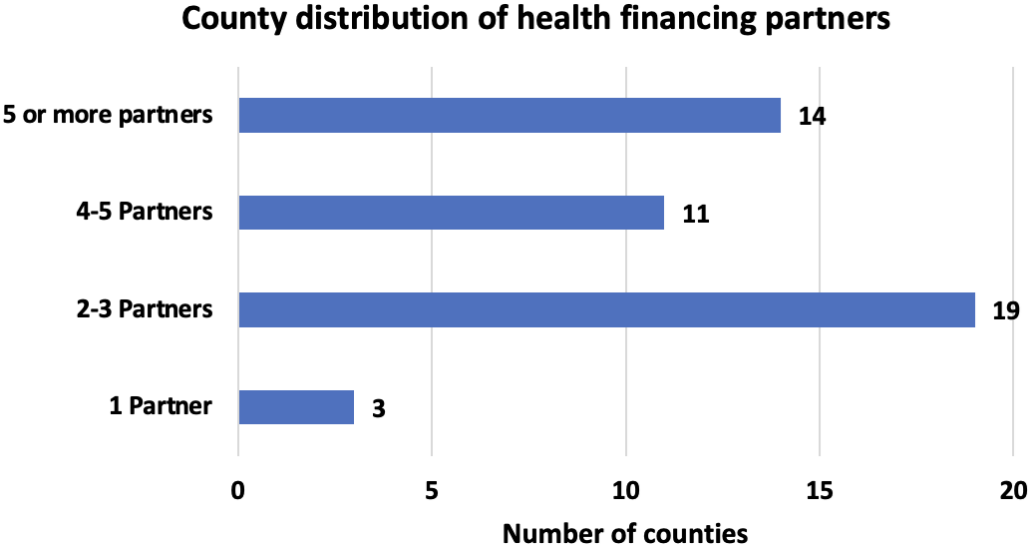
distribution of health financing partner within the counties

**Figure 4:**
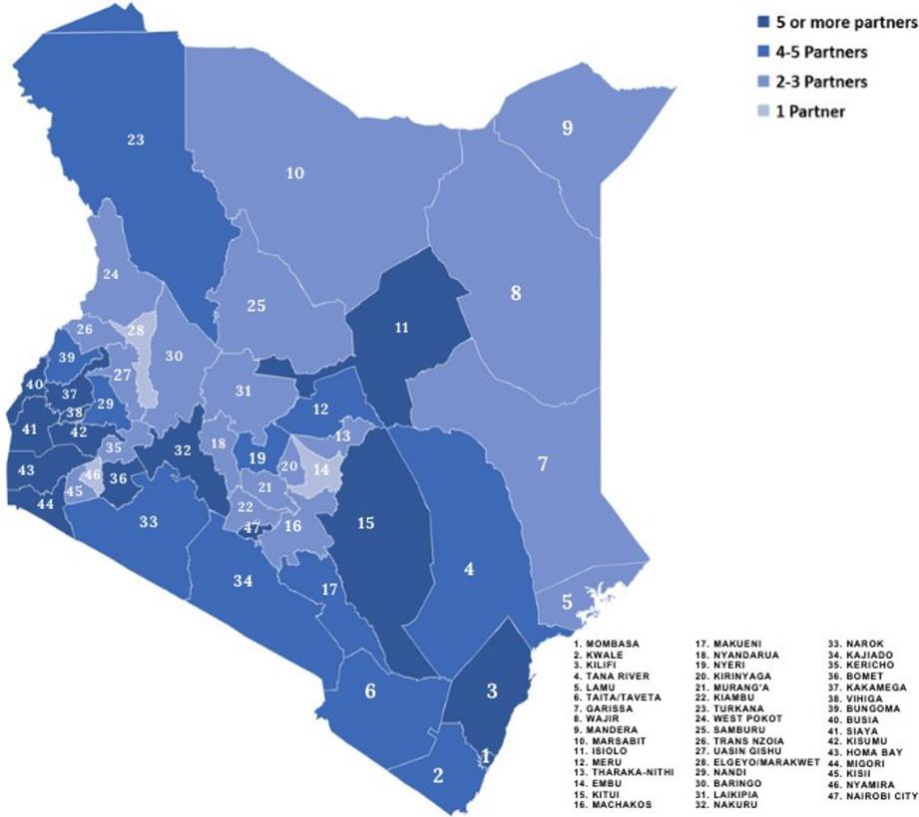
distribution of health financing partners across the counties

**Distribution of health financing partners at the counties:** most counties in Kenya had more than two partners supporting a field related to health financing, except three counties that had only one partner each (Figure 3). However, the distribution of partners varied across counties. For example, only one partner was supporting Elgeyo Marakwet, Embu and Nyamira counties compared to at least eight partners supporting Homabay, Kisumu and Kakamega counties. Fourteen (14) counties located in the western region, had at least five partners supporting health financing related work. Except Turkana and Isiolo that had more than four partners, other counties in the northern part of Kenya had between two and three partners supporting various streams of health financing work (Figure 4).

**Distribution of partner support by workstream within counties:** partners across thirty-eight (38) counties of Kenya supported health financing advocacy work, with nine counties receiving no support for this work stream. While seven counties had more than three partners supporting advocacy, and most (28) had at least two partners supporting planning. Similarly, at least two partners supported budgeting in 25 counties. The least supported streams were social accountability/reporting and resource tracking, with 19 and 21 counties receiving no partner support, respectively (Table 1).

**Table 1:** distribution of partner support by workstream in counties

		Number of counties receiving support				
Number of partners		>3	2-3	One	None	Total (counties)
Health Financing Work stream	Advocacy	7	16	15	9	38
	Planning	10	18	14	5	42
	Budgeting and costing	9	16	17	5	42
	Diagnostics	7	26	14	0	47
	Reporting and resource tracking	0	16	10	21	26
	Social accountability	2	12	14	19	28

**Qualitative findings: perspectives from senior county officials:** a total of 77 interviews were conducted with key county health officials.

**Presence of Partners at the County level:** at least one partner supported different health financing related work streams in each of the 15 counties. The main streams of work reportedly supported by partners included capacity building for planning and budgeting, advocacy and direct financing of individual programs, which included hiring of staff.

***(Note: Codes beginning with X are organisations while codes beginning with C are transcript pseudo names)***

*"We have XA1 who have helped us employ human resources and they work on HIV a great deal, XA2 also, a national government entity that is coordinating activities like the strategic plan for HIV, XA3 which is helping us in measurement and evaluation. Just recently we were coordinating with XA4 on family planning. XA5 works with us on a very special program here on training midwives to improve reproductive health. We have XA6 that has made a big difference in our level 2 and level 3 facilities because they send us money every quarter for us to be able to have those facilities running, which has assisted us because once again I want to say that the county government has not been able through our budget to give money to level 2 & 3 facilities…it is XA6 that is assisting those facilities to be operating at maximum."*C3R4

*"Yes, we have partners like XB1 who assists us in M&E and the part of planning on how to evaluate and we have also XB2 also assist us with the development of several tools and those tools help us in monitoring and implementation of whatever we have planned. We also have XA3, XB3, XB4. So, they are several among others and they carry out different activities in one way or the other and those activities help us to achieve our plans."*C6R1

**Types of health financing activities supported by partners:** most of the partners supported the counties by financing and facilitating individual programmes addressing specific community health problems. These programmes include malaria control, immunisation, nutrition, and HIV among others.

*"Yes…we have the XB5 that partners in the area of HIV. We have XB6 supporting nutrition activities then we have XC1 through transforming health services, they support in the area of reproductive health, maternal, adolescent and child health. Then we have XC2, who are also supporting some of the activities."*C10R4

Some of the partners incentivized the Community Health Volunteers CHVs) through a monthly stipend to support their programme activities at the community level, an initiative indirectly promoted other programmes within the community.

*"So, we have partners who give stipends to community health volunteers (CHVs) in 68 of 135 community units in the county. They are given a stipend of 2000 Kenya Shillings per month. So, the entry point is usually malaria…now and they are basically supporting malaria activities at the community level, but we ride on malaria activities at the community level to promote immunisation. So it is not a budget that is allocated specifically to the CHVs to promote immunisation, it is just a ride-on activity. We have others who give incentives in such a way that the CHVs are given some items to sell…they are not given money…they are just given some items to sell and out of whatever they sell, they are given some commission…"*C9R3

Three key partners offered technical support for health financing in each of the counties in the form of advocacy and capacity building for targeted health financing. The partners also regularly trained critical officials within the county health departments on programme-based budgeting, lobbying and sustainable health expenditure.

*"We have had organisations especially the ones that have done capacity building on program-based budgeting like XA3 that really helped us in capacity building on program-based budgeting…they have also incorporated XC3 in capacity building…like this thing you have seen here, the leadership development and governance is being done together with XC3 and XA3"*.C3R1

*"XB1 trained us on program-based budgeting, and they also do county accounts. So, they can tell you estimates of the expenditure on health for the county both including what people are spending out of pocket, what they are spending in the private sector, like estimates. So, when they give that, it is quite valuable information"*.C5R2

*"Yes, even from the word go XC4 were very critical in introducing program-based budgeting and as we speak even some of our officers are now in a training. We are one of the counties they are supporting under what they are calling the deep dive counties where what they were emphasising is towards universal health care…how can we reduce out of pocket expenditure…how can we make sure we absorb our budgets. They have even trained some of the officers in terms of lobbying to the county assembly health department as well as to budgeting sub-committee at the county assembly so that through that they don't slash our budget"*.C10R1

*"In some counties, partners run programs on advocacy that facilitate engagements with Members of County Assembly (MCAs) to lobby for increased resource allocation to health and to help the MCAs understand the priorities of health as they budget for the county"*.

*"There is a partner called XC5 that supported through some meetings where they shared on the issue of budgeting. They even met the MCAs to try and lobby so that even during the budget-making the MCAs should give priority to health"*.C12R1

*"XC5 at one time it was through a program on family planning, they had a program on advocacy then it allowed us to engage with MCAs. I think there was XD1 that helped us again to engage with the MCAs. So, I would say that the engagement with the MCAs has really been something that has been very consistent"*.C15R1

Partners also played a great role in supporting the planning process in counties by actively engaging in preparation and review of annual work plans (AWPs) including facilitation of meetings for developing the AWP. Some partners engage consultants to support the planning process at key stages.

*"XB1 coming on board with some support in terms of the annual work plans"*.C8R6

*"XB1 are the ones who have assisted us to come up with the AWP for the county and they have organised forums for various county stakeholders to just come together and discuss the work plan…I think we are almost finalising."*C3R5

*"XD2 supports especially the annual work plan review, they even have someone who is a person who is allocated to be part of the sector working group for budgeting so that will play a big role especially financially."*C2R2

*"XC4 and XB1 both share materials, they also go to the field and support our officers to ensure accurate data has been collected to ensure which will guide us in terms of planning yes"*.C1R3

**Challenges at the county level:** while some partners work seamlessly in cooperation with the county government, other partners prefer to plan and work independently. As a result, their activities may not meet the priority needs of the county as their work is not accounted for in the county plan. In addition, lack of coordination of partners within counties leads to duplication of activities with partners running similar programmes.

*"Others don't want to plan with you, they just come and tell you we are supporting this, others want to plan with you.…so you don't know where they planned those things but there are those who come like the XB6 and they want to plan. So, you can't bring them together when the others are having different priorities. If all of them would be thinking like XB6 we would have told others who also want to plan, so let's bring all of you together and then we come up with one plan"*.C5R2

*"So, if this thing should be streamlined and be open, straight to the point, we can be able to achieve. And then, there is also another thing of competitive activities, duplication. More than one partner is almost doing the same thing. With a clear understanding of what this partner is doing, then we can say this partner does this work on this section then other partners are barred from going to this other section. Then, other partners given a different program, then things will be able to work if they can match. Then there are single activities depending on the ability of that partner"*.C13R3

## Discussion

While all counties in Kenya had at least one partner supporting different streams of work health financing, it appeared that counties in the western region had the highest number of partners (>5) supporting health financing. Whereas there is limited data on the geographical presence of partners at the sub-national level in Kenya, the imbalance in the distribution could be attributable to the fact that most high-burden Human Immunodeficiency Virus (HIV) counties are in western Kenya [20]. A study that assessed the factors that influence non-governmental organisations (NGOs) choice of location in Kenya identified the HIV epidemic as a potential determinant for NGO placement [21].

Health financing advocacy is essential for analysing contexts and influencing focused alignment of resources for health [22]. Advocacy for improved heath financing ensures that governments meet the basic right to health of their citizens [23]. Our findings revealed that about half of the organisations provided health financing advocacy and planning in thirty-eight (38) counties. This finding indicates that advocacy targeting decision-makers at local governments has been recognized as a critical activity. Partners reported engaging in activities to identify health problems and gaps in communities through surveys and stakeholder collaboration and consultation to inform advocacy campaigns.

Stakeholders also support counties in planning by using results to come up with strategies to help counties to use the available resources efficiently. While it is the responsibility of countries to set the agenda for health financing, other stakeholders have a role to play in the process, allowing them to align their interventions with national health priorities and promote consensus on health goals. The involvement of stakeholders in planning ensures effectiveness of external aid and programmes run by implementing partners. Health planning is a necessary way in which organisations can be involved in the allocation and expenditure of government resources. Partners engaging in health planning at national and local levels should focus on exposing wastage, leakages, and bottlenecks in health financing with the aim of improving transparency and accountability [22].

Seven partners supported budgeting and costing-related works. This finding is quite concerning when compared to the findings on advocacy. Budgeting and costing are very important aspects to influence better resource allocation and prioritisation for health [24]. Given the limited resources for health, stakeholder participation limits overlapping of activities. Participatory budgeting focuses resources and funds into areas of need and distributes aspects of the budget that stakeholders can meet [25]. Participation of citizens in the formulation of budgets in the local governments has strengthened the system [26]. An earlier study in Kenya reported a persistent misalignment between planning and budgeting for health due to the dynamic nature of the planning environment [27]. Gragg proposes a data-driven forecasting approach to determine future expenditure and revenue trends to influence health budgets [28]. More support in these areas would have certainly been helpful to counties.

Five of the partners reported to support health financing diagnostics work at the county level, with all counties receiving support from at least one partner. The common health financing diagnostics conducted by partners included county budget analyses, development of budget briefs, and public expenditure reviews. Diagnostics help to identify the financing challenges facing the health system enabling policy-makers to adopt effective strategies to address the bottlenecks [29]. However, some of the partners supporting diagnostic work reported not to engage directly with counties but conducting analysis relevant to all counties. The lack of engagement implies that the 'product' may not inform or impact formulation of health financing policies at the county level. This finding highlights the need to understand how the evidence generated is cascaded to the relevant decision-makers and how it impacts how health budgets are spent.

Partners reported supporting resource tracking and reporting in 26 of the 47 counties. Each of the counties had more than three partners supporting this work stream. In Kenya, the government undertakes routine resource tracking activities for planning, even though up-to-date data on the flow of resources into public facilities is still lacking [30]. The need for an up-to-date and consistent tracking of health finances from government and external partners is increasingly attracting the interest of governments, private stakeholders, and citizens. Health expenditure tracking is necessary, especially in developing countries with limited resources, to aid the formulation of better policies and planning [31]. Resource tracking requires continuous documentation of health resources and expenditure from the government and external partners. This approach provides a clear picture of the resources that a country receives for health. For instance, a study by Ravishankar and colleagues documents the flow of global development assistance for health from organisations to low- and middle-income countries from 1990 to 2007 [32]. Morgan reiterates the importance of distinguishing national and external revenue for health at the country level is an important aspect of expenditure tracking [33].

Six partners supported social accountability work in 28 counties. Previous studies in Kenya have identified a weak capacity of communities to engage effectively in public participation forums as envisaged in the constitution [33]. This workstream entails the empowerment of communities through civic engagement to demand for accountability from their governments [34]. The fact that a significant proportion of counties do not receive technical support in social accountability implies that communities are less empowered to demand better results from the investments made at the local level. While the presence and support offered by partners were well acknowledged at the county level, the reported challenges relating to the lack of partner to partner and partner to county coordination have led to duplication of activities including misalignment with county priorities and needs. This finding is consistent with previous studies that also reported limited collaboration between healthcare partners [35,36]. The lack of coordination results in inefficiencies and inequalities in healthcare provision [37].

## Conclusion

Overall, each county had at least one partner supporting at least one health financing-related work. Planning, budgeting, and health financing advocacy were the most supported activities at the county level, with social accountability and resource tracking being the least supported activities and identified regional disparity in the distribution of partners across. Also, the study findings highlighted the challenges related to a lack of coordination of partners' support in the counties. Health sector players at both national and county levels should work towards ensuring partner coordination and collaboration to ensure that partner support at the county level is equitably distributed and addresses the needs and is well aligned with county plans for better health outcomes.

### What is known about this topic

- Apart from the government, the health sector in Kenya is financed by multiple stakeholders with private sector partners being the greatest funders of county government health systems.

### What this study adds

- This study presents the distribution of health financing partners across the country and the nature of support at the county level in Kenya;
- This study presents the county health officials' experiences and perceptions on partner support at the county level and whether it aligns with their priorities;
- These conclusions are crucial to ensure a customized approach is developed for Kenya's transition from GAVI support.
